# Traditional medicine use and associated factors in chronic patients in Jamalpur, Bangladesh: a cross-sectional study

**DOI:** 10.3389/fpubh.2025.1548728

**Published:** 2025-02-06

**Authors:** Mahmuda Akter Poli, Md Jamil Hossain, Ibrahim Kholil, Sumaya Yasmin, Bishwjit Bhowmick, Lakshmi Rani Kundu

**Affiliations:** ^1^Department of Public Health and Informatics, Jahangirnagar University, Dhaka, Bangladesh; ^2^Department of History, Jahangirnagar University, Dhaka, Bangladesh; ^3^Department of Biochemistry and Molecular Biology, Jahangirnagar University, Dhaka, Bangladesh

**Keywords:** traditional medicine, complementary and alternative medicine, modern medicine, chronic illness, Bangladesh

## Abstract

**Background:**

The history of traditional medicine is extensive. While modern medicine is commonly used to treat chronic illness, traditional medicine (TM) is gaining popularity as a healthcare practice in Bangladesh. However, evidence regarding the prevalence, patterns, and associated factors of TM use among chronic illness patients in Bangladesh remains limited. This study aimed to explore the use of traditional medicine among adults with chronic illnesses.

**Methods:**

A cross-sectional study was conducted among 518 adults with chronic illness from October to December 2023. Data was collected through face-to-face interviews. The Chi-square test was used to determine the association between categorical variables and multinomial logistic regression models to identify the factors associated with the use of traditional medicine.

**Results:**

The mean age of the participants was 43.7 (SD = 15.9) years. Among the respondents, 8.3% used traditional medicine and 5.2% used both traditional and modern medicine. Homeopathy, herbal medicine, and ayurveda were used by 69, 23, and 9%, respectively. Bivariate analysis revealed that gender (*p* = 0.014), educational qualification (*p* = 0.010), monthly income (*p* < 0.001), occupation (*p* = 0.002) and place of residence (*p* < 0.001) were statistically significantly associated with traditional medicine use. Among the respondents, 22.9% of people who used traditional medicine claimed that it was effective for disease management. Multinomial logistic regression revealed that rural individuals were seven times more likely to use TM compared to urban residents(AOR = 7.545, 95% CI: 2.933, 19.412, *p* < 0.001).

**Conclusion:**

This study revealed that individuals living in rural areas with lower monthly incomes were more likely to use traditional medicine for the treatment of chronic illnesses. Older individuals utilized it more compared to younger ones. Additionally, the study highlighted the perceived effectiveness of traditional medicine in managing chronic illnesses. These findings emphasize the need for a collaborative healthcare approach that integrates traditional medicine with modern practices to better address the diverse needs of populations.

## 1 Introduction

Traditional medicine (TM) is defined as the use of plants, animal parts, mineral, as well as spiritual therapies and techniques, for treating, diagnosing, preventing disease, or maintaining over all well-being (1). The history of traditional medicine is extensive, and millions of people around the world relying on it for treatment (2). According to the World Health Organization (WHO), around 80% of the global population depends on traditional medicine (3). Traditional medicine has been used to treat wide range of disease all over the world (4–7). Furthermore, the principles of traditional medicine have significantly influenced the development of modern medical procedures (8). Traditional medicine is widely utilized for managing and preventing chronic diseases related to lifestyle and the needs of aging populations (9). Chronic illness is a major contributor to the global disease burden, affecting health, increasing mortality, and causing economic strain, especially in low- and middle-income countries like Bangladesh (10). Every year, over 41 million people fall victim to chronic disease which account for nearly 74% of deaths worldwide (3). The primary chronic diseases include cancer, respiratory diseases, diabetes, and kidney diseases, among others (3).

Traditional medicine is considered an essential healthcare resource in low- and middle-income countries, particularly where modern medical practitioners are insufficient to meet population needs (11). Compared to modern medical practices, traditional medicine is seen as more cost-effective, readily available, and culturally acceptable (12). Moreover, The World Health Organization (WHO) has encouraged the integration of traditional medicine with contemporary treatment since 1978 (13). The use of traditional medicine (TM) varies significantly depending on the disease types, region and patient demographics, as it is influenced by cultural, historical, and personal beliefs (3, 14–16). For example, TM usage among diabetic patients ranges from 12.4 to 77.1% globally, while the rates are 36.6% among cancer patients and 34% among hypertension patients (14, 17).

A previous study conducted in Bangladesh found that 32.8% of people across all age groups used complementary and alternative medicine (18). Another study found that 35.2% of diabetic patients in Bangladesh manage their diabetes with complementary and alternative medicine (19). Various traditional medicines like Homeopathy, Ayurveda, and Unani are widely used in our country (18).

The Government of Bangladesh set a target to reduce premature mortality from non-communicable diseases (NCDs) by one-third by 2030 to achieve Sustainable Development Goal (SDG) 3.4 (20, 21). According to the World Health Organization, chronic diseases constitute around 61% of the overall burden of disease and contribute to 54% of yearly mortality in Bangladesh (22). In 2022, the World Bank estimated that only 61% of the population had access to essential healthcare services provide modern medicine, which is inadequate for 160 million people (23). The increasing burden of chronic diseases in Bangladesh emphasizes the necessity of gaining a comprehensive understanding of the treatment strategies individuals use to manage them, including their reliance on traditional medicine (TM). Our literature search found only one study on the use of complementary and alternative medicine for managing chronic illnesses (18). However, there is a significant evidence gap in our country’s understanding of the extensive use of traditional medicine in treating chronic diseases. Our geographical location and climate conditions are favorable for the growth and utilization of traditional medicine (18). Therefore, there is a huge opportunity to use traditional medicine and techniques to reduce the disease burden. Thus, the purpose of this study is to investigate the use of traditional medicine among adults with chronic illnesses. This research will contribute to building a solid body of evidence on the impact of traditional medicine in reducing the overall burden of chronic illnesses. It will also provide valuable insights for policymakers, stakeholders and government of Bangladesh to develop new strategies for integrating traditional medicine into the management of chronic illnesses and reducing mortality rates.

## 2 Materials and methods

### 2.1 Study settings and participants

A community-based cross-sectional study was conducted among individuals with chronic illnesses in the Jamalpur district between November 2023 and February 2024. Participants diagnosed with chronic illnesses and possessing valid medical records regarding their treatment process were selected for the study. After meeting all the eligibility criteria, participants were invited to participate in the study. A non-probability sampling technique (convenience sampling) was used to select 518 respondents. Data were collected through individual face-to-face interviews, and informed consent was obtained from all participants prior to the interviews.

#### 2.1.1 Inclusion criteria

1. Each respondent had a chronic illness and had been taking medication for at least 6 months.
2. The chronic illnesses included: diabetes, hypertension, cardiovascular diseases (Coronary artery disease, Heart attack and Heart failure, Strokes or Rheumatic Heart Disease), kidney diseases, cancer, allergy, tumor, migraine and others.
3. The chronic condition of the participants was diagnosed using health care facilities such as hospitals, clinics, or CAM facilities, as well as by consultation with an expert clinical practitioner or CAM practitioner.
4. Age: ≥20.
5. The participants agreed to participate in this study and completed the interview.

#### 2.1.2 Exclusion criteria

1. We omitted pregnant women from the study to reduce scientific complexity.
2. Individuals who reported pain for neuropathy or muscular pain and those who were mentally unstable.
3. Self-reported patient who did not show proper medical reports about their chronic condition and treatment practices.

### 2.2 Sample size

The sample size was calculated using the following equation:


$$n=\frac{z^{2}pq}{d^{2}};n=\frac{1.96^{2}\times 0.5\times \left(1-0.5\right)}{0.05^{2}}=384.16\approx 384$$

Here,

*n* = number of samples,

*z* = critical value of the normal distribution,

*p* = expected prevalence estimate, *q* = (1-p) = expected non-prevalence,

*d* = precision limit or proportion of sampling error.

The critical value (*z*) for a 95% confidence level is 1.96. Since there is no prior similar study, the prevalence estimate is 50%. The precision limit or proportion of sampling error (*d*) is typically considered to be 5% confidence limit. To minimize bias and misleading information, 518 samples were recruited to ensure the study’s strength.

### 2.3 Data collection tools and procedure

Five experienced interviewers who were experienced in medical data collection (nurses) were recruited for data collection. The interviewers visited the community and employed convenience sampling for data collection. Participants were first informed about the study’s purpose and procedures, and written consent was obtained from each participant. Medical records were then reviewed to confirm eligibility and verify inclusion criteria. Those who declined consent were excluded from the study. Only participants meeting all criteria proceeded to the interview stage (response rate 93%).

A semi structured questionnaire were developed using a rigorous literature review (18, 19, 24). The questionnaire was initially reviewed by an expert panel from the Department of Public Health and Informatics at Jahangirnagar University and subsequently refined based on their suggestions. Data were collected through face-to-face interviews and the questionnaire had basically three sections: A, B, and C. Section A covered socio-demographic characteristics, while Sections B and C included questions related to traditional medicine use.

### 2.4 Measures

#### 2.4.1 Socio-demographic information

Socio-demographic information included sex (male or female), age categories (<30 years, 30–50 years, 51–70 years, and > 70 years), religion (Muslim and Hindu), marital status (unmarried and married), and education levels (primary, secondary, and higher education or above). Additionally, their monthly income categories (<20,000 BDT, 20000–30,000 BDT, and > 30,000 BDT), occupations (farmers, businessmen, housewives, and office workers), and place of residence (rural and urban).

#### 2.4.2 Traditional medicine and disease related information

This section contained information about the use of traditional medicine. Participants were asked about any chronic diseases they had (diabetes, hypertension, cancer, heart disease, tumor, or another condition - please specify), how long they had been dealing with the disease, and the type of medication they used to manage it (traditional medicine, modern medicine, or a combination of both).

#### 2.4.3 Traditional medicine practice related information

Participants were asked if they used traditional medicine. If so, they were asked to specify which type (Ayurveda, herbalism, homeopathy, Unani medicine, or others) and how they learned about this treatment. Additionally, they were asked about the reasons for choosing this form of medication and whether they had ever experienced any side effects from it (yes or no).

### 2.5 Pretesting

Before conducting the final data collection, pretesting was carried out among 20 participants on the study area. Based on the insights gained from the pretesting, the questionnaire was revised and refined accordingly. Once all adjustments were made, we proceeded with the final data collection. The pretesting data were not included in the final analysis.

### 2.6 Statistical analysis

The collected data were reviewed, classified, and entered into Microsoft Excel and quality control was ensured by checking for missing and duplicate values. Descriptive statistics, including frequencies and percentages, were used to summarize categorical socio-demographic variables and continuous variables were presented as mean ± standard deviation (SD).

To examine associations between traditional medicine use and socio-demographic factors, the Chi-square (*χ*^2^) test was performed. Additionally, multinomial regression model was performed to examine the predictors on the use of traditional medicine and the combined use of traditional and modern medicine. The regression coefficients obtained from the model were presented as adjusted odds ratios (AOR) along with 95% confidence intervals (CI). A *p*-value <0.05 (two-sided) were considered statistically significant for all types of analysis. All statistical analysis were performed through IBM SPSS Statistics (version 27) and Microsoft Excel.

## 3 Results

### 3.1 Socio-demographic characteristics

Out of 518 respondents, the majority of participants (51.4%) were men, while the remaining 48.6% were female. The largest age group of respondents (48.1%) fell within the 51 to 70 years and the majority (40.7%) had completed their secondary education. Moreover, 89.2% of the participants were married, and 42.1% were housewives. Among the participants, the majority of respondents (57.9%) resided in urban areas, and approximately 52.1% had a monthly household income between 20,000 and 30,000 BDT (1 BDT = 0.0085 US$ on 9 July 2024) (Table 1).

**Table 1:** Socio-demographic status of the respondents.

Variable	Categories	Frequency*(n)*	Percentage *(%)*
Age of the participants (years)			43.70 ±15.93
Age	<30	109	21.0
	30–50	250	48.3
	51–70	133	25.7
	>70	26	5.0
Gender	Male	266	51.4
	Female	252	48.6
Religion	Islam	495	95.6
	Hindu	23	4.4
Marital status	Unmarried	56	10.8
	Married	462	89.2
Educational qualification	Primary	180	34.7
	Secondary	211	40.7
	Higher and above	127	24.5
Monthly income	<20,000	136	26.3
	20,000 to 30,000	270	52.1
	> 30,000	112	21.6
Occupation	Farmer	51	9.8
	Business	147	28.4
	Housewife	218	42.1
	Officeholder	102	19.7
Place of residence	Rural	218	42.1
	Urban	300	57.9

mean ± SD.

### 3.2 Prevalence of traditional medicine

**Figure 1:**
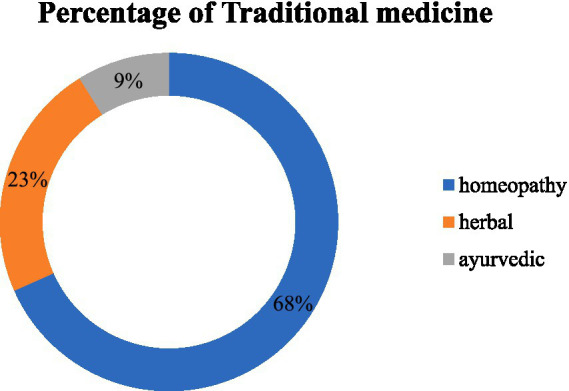
Percentages of traditional medicine use among participants.

The graph highlighted that 8.3% out of the 518 respondents used traditional medicine and 5.2% of the respondents used both traditional and modern medicine to treat their chronic illnesses. The remaining 448 respondents (86.5%) relied solely on modern medicine to manage their chronic illness. Homeopathy, herbal medicine, and ayurveda were used by 68, 23, and 9%, respectively, of those who used traditional treatment (Figure 1).

### 3.3 Percentage of the use of medication system by clinical features of participants

**Figure 2:**
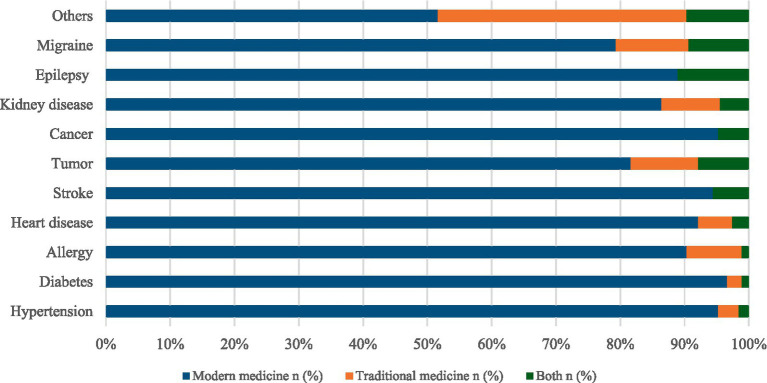
The percentage of the use of medication system by clinical features of participants.

The stacked bar highlighted the distribution of modern and traditional medicine used for chronic illnesses according to the clinical features of the respondents. The majority of participants had allergies (106), diabetes (84), tumors (62), and hypertension (60). Among the participants, those with allergies (8.3%), tumors (10.5%), and migraines (11.3%) were more likely to use traditional medicine. Conversely, the majority of respondents with diabetes (96.6%) and hypertension (95.2%) prefer modern medicine over traditional medicine (Figure 2).

### 3.4 Factors associated with the use of traditional medicine

Table 2 highlighted the various influencing factors which were associated with the use of traditional medicine. The results showed that women (10.3%) used traditional medicine significantly more than men (6.4%). There was a significant association between education and the use of traditional medicine. Respondents with higher educational qualifications were less likely to use traditional medicine, with only 2.4% reporting its use. As education levels increased, respondents were more likely to use modern medicine. Additionally, the percentage of individuals using traditional medicine decreased with higher family income (*p* < 0.001). The findings showed that the use of traditional medicine was slightly higher in low-income families (<20,000) (15.4%) compared to high-income families (>30,000) (2.7%). The use of traditional medicine was notably higher in rural areas (16.5%) than in urban areas (2.3%). Furthermore, rural residents reported a higher combined use of both traditional and modern medicines (7.3%) compared to urban residents (3.7%). This trend indicates that urbanization correlates with a decrease in the use of traditional medicine. Among different occupations, housewives reported the highest use of traditional medicine at 10.6%. Gender (*p* = 0.014), educational qualification (*p* = 0.010), monthly income (*p* < 0.001), occupation (*p* = 0.002) and place of residence (*p* < 0.001) were significantly associated with traditional medicine use (Table 2).

**Table 2:** Factors associated with the use of traditional medicine.

Variables	Categories	Modern *(%)*	Traditional *(%)*	Both *(%)*	*p*-value
Age	<30	96 (88.1%)	10 (9.2%)	3 (2.8%)	0.506
	30–50	211 (84.1%)	23 (9.2%)	16 (6.4%)	
	51–70	117 (88.0%)	9 (6.8%)	7 (5.3%)	
	>70	24 (92.3%)	1 (3.8%)	1 (3.8%)	
Gender	Male	241 (90.6)	17 (6.4%)	8 (3.0%)	**0.014**
	Female	207 (82.1%)	26 (10.3%)	19 (7.5%)	
Religion	Islam	429 (86.7%)	40 (8.1%)	26 (5.3%)	0.695
	Hindu	19 (82.6%)	3 (13.0%)	1 (4.3%)	
Marital status	Unmarried	51 (91.1%)	4 (7.1%)	1 (1.8%)	0.434
	Married	397 (85.9%)	39 (8.4%)	26 (5.6%)	
Educational qualification	Primary	145 (80.6%)	21 (11.7%)	14 (7.8%)	**0.010**
	Secondary	183 (86.7%)	19 (9.0%)	9 (4.3%)	
	Higher & above	120 (94.5%)	3 (2.4%)	4 (3.1%)	
Monthly income	<20000	100 (73.5%)	21 (15.4%)	15 (11.0%)	**<0.001**
	20000-30000	242 (89.6%)	19 (7.0%)	9 (3.3%)	
	>30000	106 (94.6%)	3 (2.7%)	3 (2.7%)	
Occupation	Farmer	45 (88.2%)	2 (3.9%)	4 (7.8%)	**0.002**
	Business	131 (89.1%)	15 (10.2%)	1 (0.7%)	
	Housewife	176 (80.7%)	23 (10.6%)	19 (8.7%)	
	Officeholder	96 (94.1%)	3 (2.9%)	3 (2.9%)	
Place of residence	Rural	166 (76.1%)	36 (16.5%)	16 (7.3%)	**<0.001**
	urban	282 (94.0%)	7 (2.3%)	11 (3.7%)	

Bold values are *P* value <0.05: statistically significant.

### 3.5 Reasons for the use of traditional medicine

**Figure 3:**
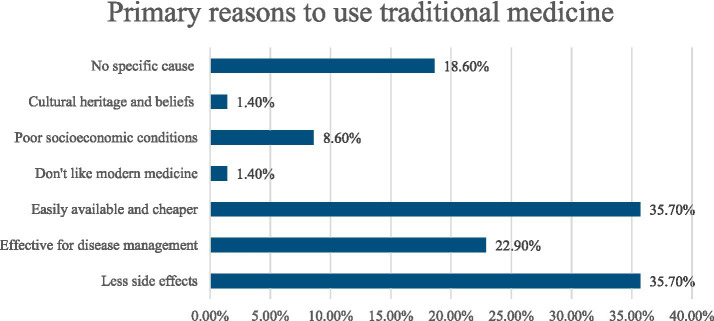
Primary reasons to use traditional medicine.

The bar chart depicted the reasons for using traditional medicine. The findings indicate that the highest percentage of people use traditional medicine because it has fewer side effects (35.7%) and is easily available and cheaper (35.7%). Additionally, 22.9% believed traditional medicine was effective for disease management, while18.6% had no specific cause. Other reasons included poor socioeconomic conditions (8.6%), a dislike for modern medicine (1.4%), cultural heritage and beliefs (1.4%), and no specific reason (18.6%) (Figure 3).

### 3.6 Source of information for traditional medicine

**Figure 4:**
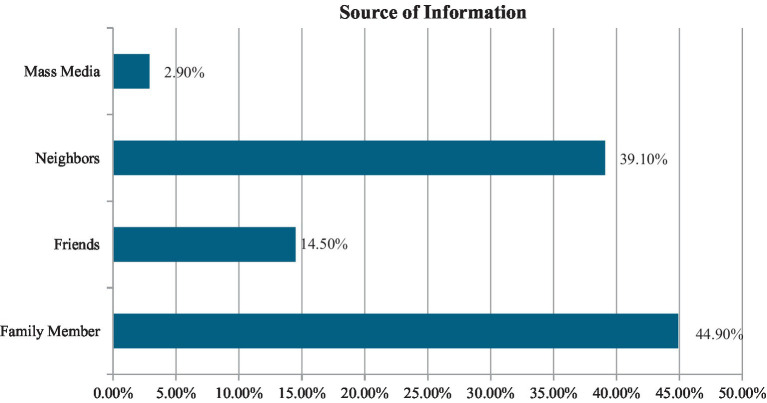
Source of information regarding traditional medicine.

This graph highlighted the source of information regarding traditional medicine. The primary sources persuading patients with chronic illnesses to use traditional medicine were families (44.9%), followed by neighbors (30.1%), friends (14.5%), and the media (2.9%) (Figure 4).

### 3.7 Multinomial logistic regression model to understand the predictors on the use of traditional medicine and the combined use of traditional-modern medicine approaches

This table demonstrated the results of a multinomial logistic regression model predicting the use of traditional medicine and the combined use of traditional and modern medicine. The findings indicate that the location of residence is statistically significant (*p* < 0.001), with rural individuals being seven times more likely to use traditional medicine than urban individuals (AOR = 7.545, 95% CI: 2.933, 19.412, *p* < 0.001). Participants aged 30 to 50 years are 2.8 times more likely to use traditional medicine compared to those over 70 years old, although the confidence interval suggests this result is not statistically reliable (AOR = 2.806, 95% CI: 0.333, 23.679, *p* = 0.343). Moreover, individuals with a monthly household income of less than 20,000 BDT are five times more likely to use both traditional and modern medicine compared to those from higher-income households, though this result also lacks statistical significance (AOR = 3.516, 95% CI: 0.661, 18.673, *p* = 0.551) (Table 3).

**Table 3:** Multinomial logistic regression model to understand the predictors on the use of traditional medicine and the combined use of traditional and modern medicine.

Variables	Categories	Traditional AOR (95% CI)	*p*-value	Both AOR (95% CI)	*p*-value
Age	<30	2.512 (0.259, 24.374)	0.427	1.231 (0.098, 15.498)	0.872
	30–50	2.806 (0.333, 23.679)	0.343	2.512 (0.300, 21.043)	0.396
	51–70	2.278 (0.253, 20.529)	0.463	2.034 (0.229, 18.073)	0.524
	>70	Reference		Reference	
Gender	Male	0.301 (0.057, 1.588)	0.157	2.246 (0.196, 25.669)	0.515
	Female	Reference		Reference	
Religion	Islam	0.900 (0.234, 3.459)	0.874	0.979 (0.115, 8.360)	0.984
	Hindu	Reference		Reference	
Marital status	Married	0.838 (0.178, 3.595)	0.824	1.515 (0.113, 20.365)	0.754
	Unmarried	Reference		Reference	
Educational qualification	Primary	2.265 (0.427, 12.011)	0.337	0.551 (0.082, 3.707)	0.540
	Secondary	2.220 (0.468, 10.525)	0.315	0.574 (0.093, 3.529)	0.549
	Higher & above	Reference		Reference	
Occupation	Farmer	0.861 (0.801, 9.103)	0.901	1.546 (0.17, 13.727)	0.696
	Business	4.093 (0.651, 25.714)	0.133	0.202 (0.016, 2.612)	0.221
	Housewife	1.229 (0.189, 8.001)	0.829	5.097(0.301, 86.269)	0.259
	Officeholder	Reference		Reference	
Monthly income	>20,000	1.579 (0.351, 7.093)	0.551	3.516(0.661, 18.673)	0.140
	20,000–30,000	1.341 (0.337, 5.348)	0.677	1.199 (0.271, 5.301)	0.811
	>30,000	Reference		Reference	
Place of residence	Rural	7.545 (2.933, 19.412)	<0.001	1.442 (0.495, 4.201)	0.503
	Urban	Reference		Reference	

## 4 Discussion

This study investigates the prevalence and predictors of traditional medicine use, as well as the factors that affect it, among adult people with chronic illness in Jamalpur District, Bangladesh. So far, this is the first study in Bangladesh primarily targeted the community population who have been suffering from chronic diseases, which is different from other studies that focused mainly on hospitalized patients.

Our study found that only 8.3% of respondents had used traditional medicine to treat chronic conditions at some point in their life. A study in Bangladesh showed a similar result, with 15.3% of respondents using exclusively complementary and alternative medicine (CAM) to manage chronic conditions (18). Furthermore, a study among diabetic patients in Bangladesh found that 35.2% utilized traditional medicine (19). This discrepancy in prevalence may be due to the fact that our study collected data from community members affected by chronic disease, while the participants in the other two studies were from specialized hospitals. In contrast, a study revealed that 56.94% of chronic illness patients in Ethiopia utilized traditional medicine (25), while in Tanzania, a staggering 70.3% relied on traditional medicine to address their chronic conditions (26). These differing prevalence rates may be due to traditional medicine playing a larger role in overall healthcare in African countries (27). According to a systematic review, between 63.8 and 91.3% of Africans with long-term chronic conditions, particularly those suffering from type 2 diabetes, use traditional treatments (28). However, many of them do not disclose this information to their doctors (29). These may because patients are concerned that reporting T&CM use may result in the termination of their traditional therapy by healthcare professionals (29). Some patients believe that revealing their use of traditional medicine might negatively impact on their relationship with their doctors and provoke an adverse reaction (30). To address this, there should be an integrated and supportive environment where patients with chronic illnesses can freely discuss their use of traditional medicine with healthcare professionals. As this type of treatment has been widely utilized in various countries (25, 26), it is essential to conduct a qualitative study to identify the barriers to using traditional medicine in our country. Moreover, further research should be conduct including a larger population across the country to accurately determine the prevalence of traditional medicine use. These findings could inform the development of public health policies that integrate TM into Bangladesh’s healthcare system, thereby addressing gaps in healthcare access, particularly in rural areas.

Our study revealed a significant difference between gender and the use of traditional medicine. We found that women were more likely to use traditional medicine than men. This aligns with similar results from previous studies, which also found that women were more likely than men to seek treatment from traditional medicine practitioners (31, 32). A study was conducted on the use of traditional healers by women in an urban Nigerian community, revealing significantly higher rates of usage among the female population (33). This may be due to traditional medicine being ingrained in cultural customs, leading women to express strong faith in its effectiveness (34). Additionally, traditional medicine is less expensive than modern medicine, which influences women prefer to use it (33, 35). Further research should explore the socio-cultural dynamics and economic factors underlying this trend to inform targeted interventions.

Additionally, our study demonstrated that patients with low levels of education were more likely to utilize TM exclusively which is similar to other studies (18, 36). This may be because educated individuals have more knowledge about modern medicine, resulting in a preference for modern treatments over traditional medicine (37, 38). However, a study discovered that both the lowest and highest income families in China showed a higher affinity for using traditional medicines (39).

In our study, 68% of participants used homeopathy, 23% used herbal medicine, and 9% used ayurveda among traditional medicine users. Similarly, previous studies consistently found that ayurveda/Unani, herbal medicine, and homeopathy were the most commonly used traditional medicines for treatment (40, 41). This may be because this variety of traditional medicine was found to be more common as it was less expensive and easily accessible than others (42). Moreover, our study illustrated that 98.6% of traditional medicine users claimed that they never experienced any side effects from using it. This finding is consistent with another study where 81% of respondents reported no side effects from using traditional medicine (43). Additionally, the main reasons for using traditional medicine were limited adverse effects, increased efficacy, and its accessibility and affordability. These factors align with findings from previous investigations (19, 36, 44). According to Basri et al. (45) 30% information regarding TM obtained from friends and 21.9% from family members. Similarly in this study, the majority of the users obtained information regarding TM from family members (44.9%), friends (14.5%), neighbors (39.1%) and media (2.9%).

From the regression model, the place of residence was statistically significant, with rural people were seven times more likely to use traditional medicine than urban people. This finding aligns with previous studies, which also showed that rural residence were more using of traditional medicine then urban people (25, 26). This might be due to the individuals in rural areas often facing limited access to modern healthcare, resulting in a greater dependence on traditional practices and herbal remedies (42, 46). Additionally, economic status play a key role in that matter for rural people, as lower-income households often prefer CAM because they are more affordable and easily available (42). However, previous studies in Bangladesh did not found any significant association between the use of traditional medicine and individuals’ place of residence (18). This difference may be due to the fact that the target population of that particular study mostly came from urban areas. Traditional medicine is widely utilized in rural part of Indonesia, where the government promotes their use to mitigate the scarcity of modern healthcare facilities (47). Similarly, Bangladesh, a lower-middle-income country, may utilize traditional medicine to help address the shortage of modern medical resources.

The study findings align with previous research conducted in Malaysia and Pakistan, indicating a significant association between socio-demographic factors, such as monthly household income, and traditional and contemporary medications use among patients with chronic illnesses (19, 48). Finally, this study highlights the importance of understanding socio-demographic and cultural factors influencing TM use among chronic illness patients in Bangladesh. Future research should focus on exploring barriers to TM use, assessing its integration into public health frameworks, and examining its role in addressing healthcare inequities.

### 4.1 Limitations of the study

Our study has certain limitations. Firstly, the use of convenience sampling introduces potential sampling biases, as the participants may not be fully representative of the broader population. Additionally, the use of a cross-sectional survey method may not allow us to identify changes in traditional medicine use over time. Secondly, while our study primarily relied on quantitative analysis, it could have been more insightful if we had also incorporated qualitative, mixed methods or explanatory analysis for providing more evidence regarding the use of TM and the impact of side effects. Thirdly, the exclusion criteria like patients have diagnosed evidence with chronic illness for at least 6 months and certain types of chronic illness in the study may reduce the generalizability of our results, as specific groups may have been unintentionally excluded from the analysis. Finally, we did not take into account the accessibility of health services, which could potentially confound the prevalence of traditional medicine use.

## 5 Conclusion

The study found that 10.3% of women and 6.8% of men exclusively used traditional medicine (TM). The use of traditional medicine was statistically associated with the respondent’s gender, educational qualification, monthly income, occupation, and place of residence. The results showed that individuals from rural areas and those with lower family income were more likely to use traditional medicine. Additionally, users claimed that traditional medicine was effective for managing chronic diseases. So, individuals should consider incorporating traditional medicine into their overall healthcare approach. This study emphasizes the need for a comprehensive and collaborative approach between traditional medical practitioners and modern healthcare professionals to achieve better healthcare outcomes, particularly in underserved rural areas in Bangladesh. Future healthcare policies should focus on developing guidelines for the combined use of traditional and modern medicine, promoting research to evaluate the efficacy of traditional therapies, and ensuring the affordability and accessibility. Therefore, a comprehensive and balanced approach to healthcare may be attained by cooperative efforts between traditional medical practitioners and contemporary healthcare specialists.

## Data Availability

The raw data supporting the conclusions of this article will be made available by the authors, without undue reservation.

## References

[1] Fokunang CN Ndikum V Tabi OY Jiofack RB Ngameni B Guedje NM. Traditional medicine: past, present and future research and development prospects. Afr J Tradit Complement Altern Med. (2011) 8:284–95. doi: 10.4314/ajtcam.v8i3.65276, PMID: 22468007 PMC3252219

[2] WHO. Traditional medicine has a long history of contributing to conventional medicine and continues to hold promise. (2024). Available at: https://www.who.int/news-room/feature-stories/detail/traditional-medicine-has-a-long-history-of-contributing-to-conventional-medicine-and-continues-to-hold-promise

[3] WHO. Noncommunicable diseases. (2023). Available at: https://www.who.int/news-room/fact-sheets/detail/noncommunicable-diseases

[4] Nigussie S Godana A Birhanu A Abdeta T Demeke F Lami M. Practice of traditional medicine and associated factors among residents in eastern Ethiopia: a community-based cross-sectional study. Front Public Heal. (2022) 10:1–8. doi: 10.3389/fpubh.2022.915722, PMID: 35774577 PMC9237408

[5] Ijaz N Hunter J Grant S Templeman K. Protocol for a scoping review of traditional medicine research methods, methodologies, frameworks and strategies. Front Med. (2024) 11:1–14. doi: 10.3389/fmed.2024.1409392, PMID: 39050530 PMC11267516

[6] Ali MS Getaneh Mekonen E Workneh BS. Spatial variation and determinants of traditional birth attendants utilization among women of reproductive age in Ethiopia: spatial and multilevel analysis study. SAGE Open Med. (2024) 12:12. doi: 10.1177/20503121241282257, PMID: 39346618 PMC11437578

[7] Aferu T Mamenie Y Mulugeta M Feyisa D Shafi M Regassa T. Attitude and practice toward traditional medicine among hypertensive patients on follow-up at Mizan–Tepi university teaching hospital, Southwest Ethiopia. SAGE Open Med. (2022) 10:10. doi: 10.1177/20503121221083209, PMID: 35310931 PMC8928343

[8] Yuan H Ma Q Ye L Piao G. The traditional medicine and modern medicine from natural products. Molecules. (2016) 21:1–18. doi: 10.3390/molecules21050559, PMID: 27136524 PMC6273146

[9] WHO. WHO global report on traditional and complementary medicine 2019. World Health Organization, editor. WHO. (2019) 1–226. Available at: https://www.who.int/publications/i/item/978924151536

[10] Mahumud RA Gow J Mosharaf MP Kundu S Rahman MA Dukhi N. The burden of chronic diseases, disease-stratified exploration and gender-differentiated healthcare utilisation among patients in Bangladesh. PLoS One. (2023) 18:e0284117. doi: 10.1371/journal.pone.0284117, PMID: 37130132 PMC10153713

[11] Li S Odedina S Agwai I Ojengbede O Huo D Olopade OI. Traditional medicine usage among adult women in Ibadan, Nigeria: a cross-sectional study. BMC Complement Med Ther. (2020) 20:93. doi: 10.1186/s12906-020-02881-z, PMID: 32192455 PMC7083039

[12] Sato A. Revealing the popularity of traditional medicine in light of multiple recourses and outcome measurements from a user’s perspective in Ghana. Health Policy Plan. (2012) 27:625–37. doi: 10.1093/heapol/czs010, PMID: 22345671

[13] WHO. Traditional medicine strategy (2002–2005). (2002). Available at:https://www.who.int/publications/i/item/WHO-EDM-TRM-2002.1

[14] Aboufaras M Selmaoui K Najib R Lakhdissi A Ouzennou N. Predictors of herbal medicine use among cancer patients. J Cancer Res Clin Oncol. (2023) 149:4991–5005. doi: 10.1007/s00432-022-04451-x, PMID: 36318333 PMC11798265

[15] Grace R Vaz J Da Costa J. Traditional medicine use in Timor-Leste. BMC Complement Med Ther. (2020) 20:165. doi: 10.1186/s12906-020-02912-9, PMID: 32493305 PMC7268523

[16] Peltzer K Pengpid S. Utilization and practice of traditional/complementary/alternative medicine (T/CAM) in southeast Asian nations (ASEAN) member states. Stud Ethno-Medicine. (2015) 9:209–18. doi: 10.1080/09735070.2015.11905437

[17] Traoré F Bamba KD Ngoran YNK Koffi F Mottoh MP Esaie S. Traditional medicine followed at the heart Institute of Abidjan. World J Cardiovasc Dis. (2017) 7:292–8. doi: 10.4236/wjcd.2017.79027

[18] Shahjalal M Chakma SK Ahmed T Yasmin I Mahumud RA Hossain A. Prevalence and determinants of using complementary and alternative medicine for the treatment of chronic illnesses: a multicenter study in Bangladesh. PLoS One. (2022) 17:e0262221. doi: 10.1371/journal.pone.0262221, PMID: 34986159 PMC8730415

[19] Rafi MA Azad DT Bhattacharjee M Rahman N Mubin KA Rahman MA. A hospital-based study on complementary and alternative medicine use among diabetes patients in Rajshahi, Bangladesh. BMC Complement Med Ther. (2020) 20:219. doi: 10.1186/s12906-020-03021-3, PMID: 32660539 PMC7359228

[20] Islam K Huque R Saif-Ur-Rahman KM Ehtesham Kabir ANM Enayet Hussain AHM. Implementation status of non-communicable disease control program at primary health care level in Bangladesh: findings from a qualitative research. Public Heal Pract. (2022) 3:100271. doi: 10.1016/j.puhip.2022.100271, PMID: 36101774 PMC9461504

[21] WHO. The Global Health Observatory. (2024). Available at: https://www.who.int/data/gho/data/themes/topics/indicator-groups/indicator-group-details/GHO/sdg-target-3.4-noncommunicable-diseases-and-mental-health

[22] WHO. Global health risks: Mortality and burden of disease attributable to selected major risks. Geneva PP - Geneva: World Health Organization (2009).

[23] TBS Report. The business standard. Ensuring equitable access, quality healthcare still a challenge in Bangladesh: experts. (2024). Available at: https://www.tbsnews.net/bangladesh/health/ensuring-equitable-access-quality-healthcare-still-challenge-bangladesh-experts

[24] Mohsin FM Das GS Hasan S Mahmud ST Yasmin I Hossain MA. Complementary and alternative medicine use by Bangladeshi adult patients with diabetes and hypertension: a multicenter study. F1000Research. (2023) 12:1063. doi: 10.12688/f1000research.139803.1

[25] Tassew WC Assefa GW Zeleke AM Ferede YA. Prevalence and associated factors of herbal medicine use among patients living with chronic disease in Ethiopia: a systematic review and meta-analysis. Metab Open. (2024) 21:100280. doi: 10.1016/j.metop.2024.100280, PMID: 38455230 PMC10918421

[26] Stanifer JW Lunyera J Boyd D Karia F Maro V Omolo J. Traditionalmedicine practices among communitymembers with chronic kidney disease innorthern Tanzania: an ethnomedical survey. BMC Nephrol. (2015) 16:170. doi: 10.1186/s12882-015-0161-y, PMID: 26499070 PMC4619231

[27] Sankaramourthy D Subramanian K Sadras SR. Safety and regulatory issues on traditional medicine entrusted drug discovery. Evid Based Valid Tradit Med A Compr Approach. (2021) 589–603. doi: 10.1007/978-981-15-8127-4_28

[28] Ekpor E Osei E Akyirem S. Prevalence and predictors of traditional medicine use among persons with diabetes in Africa: a systematic review. Int Health. (2024) 16:252–60. doi: 10.1093/inthealth/ihad080, PMID: 37706354 PMC11062204

[29] Hassali MA Farooqui M Saleem F Roslan MN. Disclosing Traditional & Complementary Medicine (T&CM) use to the health care providers: a qualitative study among thalassemia patients in Malaysia. Value Heal. (2017) 20:A726. doi: 10.1016/j.jval.2017.08.1965, PMID: 39870102

[30] Kelak JA Cheah WL Safii R. Patient’s decision to disclose the use of traditional and complementary medicine to medical doctor: a descriptive phenomenology study. Evidence-Based Complement Altern Med. (2018) 2018:4735234. doi: 10.1155/2018/4735234, PMID: 29636778 PMC5832099

[31] James PB Gyasi RM Kasilo OMJ Wardle J Bah AJ Yendewa GA. The use of traditional medicine practitioner services for childhood illnesses among childbearing women: a multilevel analysis of demographic and health surveys in 32 sub-Saharan African countries. BMC Complement Med Ther. (2023) 23:137. doi: 10.1186/s12906-023-03972-3, PMID: 37120536 PMC10148432

[32] Wardani WT Wahyudi A. Gambaran pengetahuan dan pengalaman penggunaan obat tradisional pada pelajar SLTA. Heal Sci Pharm J. (2023) 7:109–17. doi: 10.32504/hspj.v7i2.780

[33] Goodman OO Adejoh SO Adeniran A Emechebe AC Kuyinu YA. We love orthodox medicine but still use our ‘Elewe omo’: utilization of traditional healers among women in an urban community in Nigeria. J Fam Med Prim Care. (2022) 11:215–23. doi: 10.4103/jfmpc.jfmpc_1302_21, PMID: 35309609 PMC8930113

[34] Murugan P Yared P. Beliefs and practices of traditional medicine towards women’s reproductive healthcare: evidences from Wolaytta zone, Ethiopia. Ital Sociol Rev. (2018) 8:157. doi: 10.13136/isr.v8i2.153

[35] Shewamene Z Dune T Smith CA. Use of traditional and complementary medicine for maternal health and wellbeing by African migrant women in Australia: a mixed method study. BMC Complement Med Ther. (2020) 20:1–12. doi: 10.1186/s12906-020-2852-6, PMID: 32070348 PMC7076811

[36] Abdullahi AA. Trends and challenges of traditional medicine in Africa. African J Tradit Complement Altern Med. (2011) 8:115–23. doi: 10.4314/ajtcam.v8i5SS.5 PMC3252714 22754064

[37] Przybylska D Drop B Przybylski P Drop K. Health education as an important tool in the healthcare system. Zdr Publiczne. (2014) 124:145–7. doi: 10.2478/pjph-2014-0032, PMID: 39513035

[38] Tabi MM Powell M Hodnicki D. Use of traditional healers and modern medicine in Ghana. Int Nurs Rev. (2006) 53:52–8. doi: 10.1111/j.1466-7657.2006.00444.x, PMID: 16430761

[39] Xin B Mu S Tan T Yeung A Gu D Feng Q. Belief in and use of traditional chinese medicine in shanghai older adults: a crosssectional study. BMC Complement Med Ther. (2020) 20:128. doi: 10.1186/s12906-020-02910-x, PMID: 32345283 PMC7189641

[40] Karmakar P Islam MM Kibria MG Hossain MS Sattar MM. Prevalence, belief and awareness of preferring traditional healthcare system in urban and rural people of Noakhali district, Bangladesh. Int Curr Pharm J. (2012) 1:229–34. doi: 10.3329/icpj.v1i9.11611

[41] Kala CP. Traditional health care systems and herbal medicines in India. Eur J Environ Public Heal. (2017) 1:1–6. doi: 10.20897/ejeph.201703

[42] Sojasi Qidari H Afshar Z. Assessment of trends to traditional treatments by using herbal medicine in rural areas. Med Hist. (2017) 7:185–220.

[43] Uddin MZ Hassan MA Sultana M. Ethnobotanical survey of medicinal plants in Phulbari Upazila of Dinajpur District Bangladesh. Bangladesh J Plant Taxon. (1970) 13:63–8. doi: 10.3329/bjpt.v13i1.596

[44] Peltzer K Pengpid S. The use of herbal medicines among chronic disease patients in Thailand: a cross-sectional survey. J Multidiscip Healthc. (2019) 12:573–82. doi: 10.2147/JMDH.S212953, PMID: 31413584 PMC6661386

[45] Basri NF Ramli AS Mohamad M Kamaruddin KN. Traditional and complementary medicine (TCM) usage and its association with patient assessment of chronic illness care (PACIC) among individuals with metabolic syndrome in primary care. BMC Complement Med Ther. (2022) 22:1–15. doi: 10.1186/s12906-021-03493-x, PMID: 35027058 PMC8759276

[46] Ansari MA Khan Z. Use of local health traditions for prevention and cure of diseases by households in northern India. MOJ Public Heal. (2018) 7:393–8. doi: 10.15406/mojph.2018.07.00273

[47] Suharmiati S Laksono AD Nantabah ZK Kristiana L. Urban-rural disparities in traditional health service use in Indonesia: a cross-sectional study. J Southwest Jiaotong Univ. (2023) 58:231–36. doi: 10.35741/issn.0258-2724.58.3.32

[48] Shaikh BT Hatcher J. Complementary and alternative medicine in Pakistan: prospects and limitations. Evidence-Based Complement Altern Med. (2005) 2:139–42. doi: 10.1093/ecam/neh088, PMID: 15937553 PMC1142200

